# Biochemical mechanisms underpinning impaired endogenous nitric oxide formation with the use of proton pump inhibitors

**DOI:** 10.4103/mgr.MEDGASRES-D-25-00170

**Published:** 2026-05-15

**Authors:** Macario A. Rebelo, Carla Fiama de Azevedo Medeiros, Heitor Moreno, Jose E. Tanus-Santos

**Affiliations:** Department of Translational Medicine, State University of Campinas, Campinas, SP, Brazil; Department of Pharmacology, Ribeirao Preto Medical School, University of São Paulo, Ribeirão Preto, SP, Brazil; Department of Internal Medicine, Faculty of Medical Sciences, State University of Campinas, Campinas, SP, Brazil

##  

**Nitric oxide (NO) and blood pressure regulation:** Impaired NO bioavailability plays a major role in a variety of diseases, particularly cardiovascular diseases and their complications. Therefore, supplementing endogenous NO formation is an important pharmacological strategy to treat those conditions.^1^ Conversely, identifying mechanisms that may further impair NO bioavailability may help improve therapy and prevent complications in such patients. In this regard, accumulating evidence indicates that the long-term use of drugs commonly prescribed to suppress gastric acid secretion, namely proton pump inhibitors (PPIs), may impair normal endothelial function and eventually lead to hypertension.

Strategies that promote NO accumulation in tissues to restore NO bioavailability may be highly useful for reestablishing homeostasis.^2^ In this article, we discuss the biochemical mechanisms underlying impaired endogenous enzymatic and non-enzymatic NO formation associated with PPI use, as well as those affecting a complementary pathway of NO formation, which involves the reduction of nitrate to nitrite and its subsequent bioactivation to NO in the stomach. We also explain how acid suppression impairs both processes and discuss the implications for the pathogenesis and treatment of hypertension.

**PPIs inhibit NO synthase (NOS) activity:** The widespread use of gastric acid secretion inhibitors, such as PPIs, has recently become a focus of concern, as mounting evidence indicates that these drugs may not be devoid of important side effects. They are widely prescribed for gastroesophageal reflux disease, peptic ulcers, and other acid-related disorders. In particular, PPIs (lansoprazole, esomeprazole, rabeprazole, omeprazole, tenatoprazole, and pantoprazole) have been shown to affect endogenous NO production by inhibiting endothelial NOS, which synthesizes NO from L-arginine.^3^ PPI-induced inhibition of endogenous NO production is explained by the suppression of dimethylarginine dimethylaminohydrolase-1 (**Figure 1**), an enzyme involved in the degradation of asymmetric dimethylarginine, a NOS inhibitor. The resulting increase in asymmetric dimethylarginine concentrations competitively inhibits NOS and impairs NO formation.^3^ Therefore, the chronic use of PPIs may impair NO activity, promote oxidative stress in the vasculature, impair vascular smooth muscle relaxation, and contribute to increased vascular resistance and remodeling,^4^ a hallmark of hypertension.

**Figure 1:**
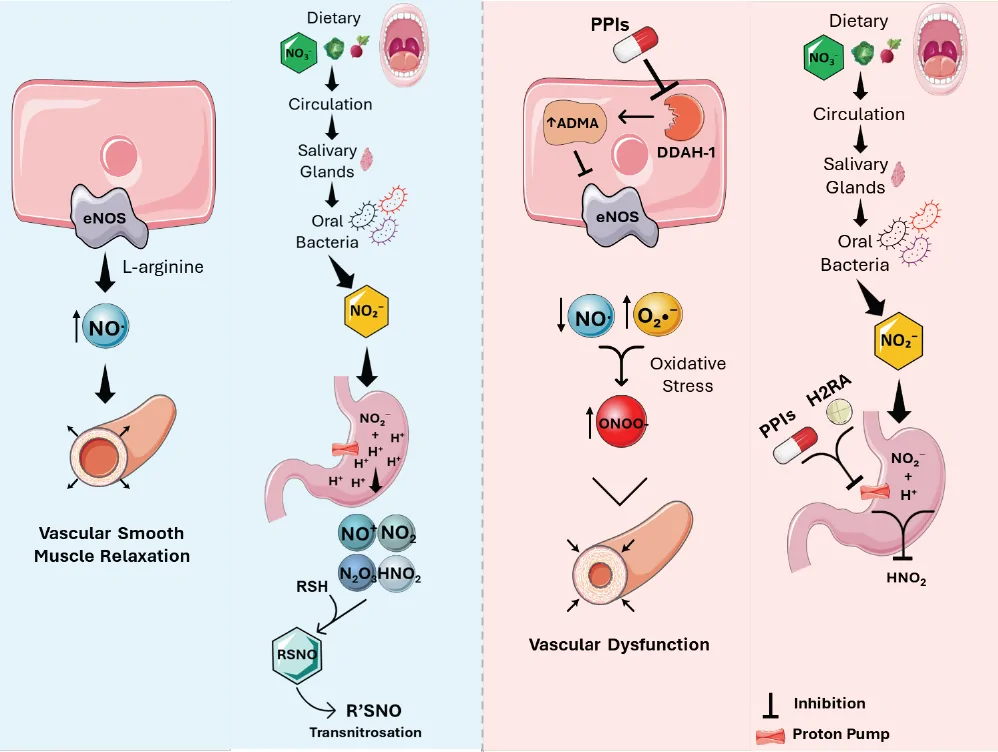
Mechanisms involved in endogenous NO formation as a result of NO synthase activity or activation of nitrate-nitrite-NO pathway. Both pathways are summarized on the left (blue background), whereas the interference of PPIs or H2RAs is shown on the right (red background). Adapted from Servier Medical Art (https://smart.servier.com/), licensed under CC BY 4.0 (https://creativecommons.org/licenses/by/4.0/). ADMA: Asymmetric dimethylarginine; DDAH1: dimethylarginine dimethylaminohydrolase 1; eNOS: endothelial nitric oxide synthase; H2RA: histamine H_2_ receptor antagonist; HNO_2_: nitrous acid; NO: nitric oxide; NO_2_^−^: nitrite; NO_3_^−^: nitrate; PPI: proton pump inhibitor; RSH: thiol; RSNO: nitrosothiol.

**Nitrate and nitrite bioactivation depend on low gastric pH:** Although inhibition of NOS activity is highly relevant, there is now evidence that PPIs may contribute to impaired NO activity and hypertension by mechanisms beyond decreased enzymatic NO synthesis. Indeed, it is now well established that dietary nitrate (NO_3_^–^) and nitrite (NO_2_^–^) serve as alternative sources of NO, and PPIs, as well as other drugs, may severely interfere with their bioactivation to NO (**Figure 1**). The so-called nitrate-nitrite-NO pathway is now recognized as a complementary mechanism contributing to NO signaling and vascular function, particularly under disease conditions. In fact, NO is usually oxidized to nitrite and nitrate, which were once considered inert byproducts of NO metabolism. However, over the past two decades, both anions have been widely recognized as important sources of NO independent of NOS. They can be recycled back to NO and other bioactive nitrogen oxides and, for this reason, have been investigated as pharmacological tools to supplement endogenous NO formation.^1^

Nitrate and nitrite bioactivation to NO and other nitroxides involves a complex biological circuit, now known as the enterosalivary cycle of nitrate, which may be severely affected by PPIs because these drugs increase gastric pH.^1^ After the ingestion of food rich in nitrate, this anion is physiologically absorbed in the gastrointestinal tract into the circulation, and approximately 75% of dietary nitrate is excreted unchanged in the urine. The residual nitrate is taken up by the salivary glands and secreted with the saliva into the oral cavity, where commensal bacteria promote nitrate reduction to nitrite as a result of bacterial nitrate-reductase activity. The saliva containing increased concentrations of nitrite is then swallowed and readily protonated in the acidic environment of the stomach, generating nitrous acid, which decomposes further to NO and other bioactive nitrogen oxides, including HNO_2_, N_2_O_3_, and NO_2_. These molecules function as [NO^+^] carriers and may chemically react with deprotonated thiols to form S-nitrosothiols.^1^ This mechanism of nitrate bioactivation to nitrite and other NO-related species involves a series of intricate biochemical transformations that notably depend on the acidic environment of the stomach. Therefore, the stomach could be considered a bioreactor converting dietary nitrate and nitrite into bioactive NO species. However, central to this pathway is the formation of S-nitrosothiols, which are carriers of NO and may also promote very important post-translational modifications in protein targets, including pharmacological receptors in the vasculature, by inducing transnitrosation reactions (the transfer of NO from an RSNO to another R´SNO; **Figure 1**), especially on their cysteine residues.^1^

**PPIs and other inhibitors of gastric acid secretion inhibit the responses to nitrite or nitrate:** The drugs most frequently prescribed to suppress gastric acid secretion include PPIs and histamine H_2_ receptor antagonists (H2RAs). Recent studies have convincingly shown that the increases in gastric pH associated with the use of these drugs may have a major impact on the physiological responses to dietary nitrite or nitrate.^5^ It is remarkable that acute treatment with oral nitrite resulted in dose-dependent decreases in arterial blood pressure that were severely attenuated in animals previously treated with omeprazole, a commonly used PPI.^5^ Consistent with these initial results, chronic treatment with oral nitrate or nitrite also resulted in major antihypertensive effects, which were blunted by simultaneous treatment with omeprazole.^1^,^6^ Importantly, the antihypertensive responses to orally administered nitrite or nitrate were positively associated with increases in total plasma S-nitrosothiol concentrations and independent of increases in circulating nitrite levels.^1^ These findings suggest that the gastric formation of S-nitrosothiols is a critical mechanism involved in the blood pressure responses to oral nitrite or nitrate administration. Further supporting this idea, simultaneous treatment with omeprazole blunted the increases in circulating S-nitrosothiols associated with nitrite or nitrate treatment. Very similar findings were reported when ranitidine (an H2RA) was used to suppress gastric acid secretion.^6^ Treatment with this drug blunted both the hypotensive responses to oral nitrite treatment and the increases in circulating S-nitrosothiols. Moreover, when sodium acetate buffer was used to mimic the changes in gastric pH caused by omeprazole or ranitidine, the same alterations were observed in the responses to oral nitrite treatment compared with those found when the PPI or the H2RA were tested.^6^ Together, these studies provide strong experimental evidence that drugs increasing gastric pH disrupt the enterosalivary cycle of nitrate by interfering with the chemical reactions that physiologically take place in the stomach and result in the formation of S-nitrosothiols associated with hypotensive responses. Curiously, the mechanism described above links gastric physiology directly to systemic vascular regulation, as recently shown in studies demonstrating that omeprazole inhibited the gastric formation of S-nitrosothiols and the nitrosation of vascular targets, including calcium/calmodulin-dependent protein kinase II (mediating α1-adrenergic receptor signaling)^7^ and protein kinase C (mediating angiotensin II vasoconstriction).^8^

These observations offer a new mechanistic perspective on the effects of both nitrite and nitrate and have major implications, particularly for patients who are prescribed drugs that inhibit gastric acid secretion. This perspective may be especially relevant for patients with subclinical cardiovascular diseases or for those exposed to a variety of cardiovascular risk factors known to deplete cardiovascular NO bioavailability.

Giving further support to the idea that S-nitrosothiol formation is important for the physiology of blood pressure regulation, a recent study showed that treatment of overweight women for 14 days with dietary nitrate improved endothelial function and lowered blood pressure in association with increases in circulating S-nitrosothiol concentrations.^9^ However, these beneficial effects were not observed in overweight women who had their gastrointestinal anatomy modified by prior bariatric Roux-en-Y gastric bypass surgery.^9^ This is explained by major changes induced by Roux-en-Y gastric bypass surgery in gastric pH, which disrupt the formation of S-nitrosothiols stimulated by increased dietary nitrate content. This surgery produces alterations in gastric pH similar to those caused by inhibitors of gastric acid secretion discussed above and prevents both nitrate and nitrite from exerting their antihypertensive effects. It remains to be determined whether impaired S-nitrosothiol formation after this surgery triggers or exacerbates hypertension in patients who have undergone such surgical intervention.

The interactions of inhibitors of gastric acid secretion with the intestinal microbiome are relevant and should be explored to improve our understanding of how nitrate and nitrite bioactivation takes place. Even though acidic gastric pH is important for the responses to oral nitrite, recent findings suggest that using an enteric-coated formulation of nitrite (allowing nitrite release only in the intestine) could result in greater increases in circulating NO metabolite concentrations and improved responses to oral nitrite.^10^ Whether acid-suppressive drugs that act in the stomach also exert such effects in the intestine remains to be determined.

**PPIs and other inhibitors of gastric acid secretion may cause long-term consequences that contribute to hypertension:** It is now clear that the impaired endogenous formation of NO and the bioactivation of nitrate/nitrite to NO associated with the chronic use of PPIs and H2RAs may directly promote hypertensive mechanisms by interfering with NO-mediated vasorelaxation.^1^,^6^ However, there is evidence that such drugs may also contribute to long-term vascular remodeling as a result of pro-oxidant alterations in the vasculature, which may activate a group of enzymes involved in extracellular matrix recycling, the matrix metalloproteinases (MMPs).^4^ Increased oxidative stress associated with the use of PPIs and H2RAs may be caused by enhanced superoxide formation, and this mechanism usually decreases NO bioavailability because NO reacts promptly with superoxide to form peroxynitrite.^4^ These chemical reactions promote the activation of MMPs and may underlie the vascular remodeling associated with the use of PPIs and H2RAs, as recently suggested.^4^

While numerous studies have highlighted significant concerns about NO depletion and cardiovascular complications associated with the use of PPIs, potentially elevating cardiovascular risk, fewer studies have shown similar effects with H2RAs. Therefore, the use of H2RAs has been viewed to potentially circumvent the safety concerns surrounding the long-term use of PPIs. However, recent findings suggest that chronic treatment with the H2RA ranitidine leads to vascular changes akin to those seen with omeprazole.^4^ Specifically, ranitidine was found to impair vascular function, as assessed by an NO-mediated response (vasorelaxation induced by acetylcholine), and to increase vascular MMP-2 gelatinolytic activity, triggering vascular remodeling, all of which occurred in association with increased vascular reactive oxygen species production.^4^ These effects of H2RAs closely mirrored those documented with PPIs, suggesting that the heightened cardiovascular risk may be a broader consequence of intragastric acid suppression, regardless of the drug class used.

**Conclusion:** The chronic use of drugs that inhibit gastric acid secretion, particularly PPIs and H2RAs, poses significant concerns as a contributor to impaired NO bioactivity, thus increasing cardiovascular risk, especially in patients with other risk factors for cardiovascular disease. By interfering with both enzymatic NO synthesis and the gastric bioactivation of dietary nitrate and nitrite, particularly the formation of S-nitrosothiols, these drugs have the potential to compromise key physiological pathways involved in blood pressure regulation, thereby promoting the development of hypertension. The resultant decrease in NO bioavailability, compounded by oxidative stress and extracellular matrix remodeling via MMP activation, in addition to other mechanisms, may directly promote long-term vascular dysfunction and facilitate pro-hypertensive mechanisms. These findings underscore the need for careful evaluation of the cardiovascular consequences of prolonged acid suppression therapy, particularly in populations already vulnerable to endothelial dysfunction or hypertension. Further research is warranted to validate these recent findings in the clinical setting and fully elucidate their implications. It is important to explore safer pharmacological alternatives or mitigation strategies for patients requiring long-term acid suppression. One straightforward approach to mitigate the hypertension risk associated with the use of inhibitors of gastric acid secretion is to minimize unnecessary or prolonged use of these drugs, especially in patients with cardiovascular comorbidities. Where acid suppression is essential, strategies to preserve or restore gastric NO bioactivation pathways merit exploration.

*The authors declare that no generative AI was used in the preparation of this manuscript*.
